# Focused ultrasound excites action potentials in mammalian peripheral neurons in part through the mechanically gated ion channel PIEZO2

**DOI:** 10.1073/pnas.2115821119

**Published:** 2022-05-17

**Authors:** Benjamin U. Hoffman, Yoshichika Baba, Stephen A. Lee, Chi-Kun Tong, Elisa E. Konofagou, Ellen A. Lumpkin

**Affiliations:** ^a^Department of Physiology & Cellular Biophysics, Columbia University, New York, NY 10032;; ^b^Program in Neurobiology & Behavior, Columbia University, New York, NY 10032;; ^c^Department of Medicine, University of California, San Francisco, CA 94143;; ^d^Department of Molecular and Cell Biology, University of California, Berkeley, CA 94720;; ^e^Department of Biomedical Engineering, Columbia University, New York, NY 10032

**Keywords:** peripheral nerve stimulation, neuromodulation, ultrasound, somatosensory, PIEZO2

## Abstract

Neurons of the peripheral nervous system (PNS) are tasked with diverse roles, from encoding touch, pain, and itch to interoceptive control of inflammation and organ physiology. Thus, technologies that allow precise control of peripheral nerve activity have the potential to regulate a wide range of biological processes. Noninvasive modulation of neuronal activity is an important translational application of focused ultrasound (FUS). Recent studies have identified effective strategies to modulate brain circuits; however, reliable parameters to control the activity of the PNS are lacking. To develop robust noninvasive technologies for peripheral nerve modulation, we employed targeted FUS stimulation and electrophysiology in mouse ex vivo skin-saphenous nerve preparations to record the activity of individual mechanosensory neurons. Parameter space exploration showed that stimulating neuronal receptive fields with high-intensity, millisecond FUS pulses reliably and repeatedly evoked one-to-one action potentials in all peripheral neurons recorded. Interestingly, when neurons were classified based on neurophysiological properties, we identified a discrete range of FUS parameters capable of exciting all neuronal classes, including myelinated A fibers and unmyelinated C fibers. Peripheral neurons were excited by FUS stimulation targeted to either cutaneous receptive fields or peripheral nerves, a key finding that increases the therapeutic range of FUS-based peripheral neuromodulation. FUS elicited action potentials with millisecond latencies compared with electrical stimulation, suggesting ion channel–mediated mechanisms. Indeed, FUS thresholds were elevated in neurons lacking the mechanically gated channel PIEZO2. Together, these results demonstrate that transcutaneous FUS drives peripheral nerve activity by engaging intrinsic mechanotransduction mechanisms in neurons [B. U. Hoffman, PhD thesis, (2019)].

## Significance

Modulation of peripheral nervous system (PNS) activity has shown promise in treating a wide range of diseases, from epilepsy to rheumatoid arthritis. Clinically, stimulation of nerves is most commonly delivered through invasive and risk-laden surgical electrode placement. Noninvasive technologies for PNS modulation can both increase safety and expand modulation application to various disease stages. Recent studies have revealed the therapeutic potential of noninvasive neuromodulation of brain circuits with ultrasound. This study identifies reliable protocols and molecular mechanisms for stimulating action potentials from individual peripheral neurons in the mammalian nervous system. These findings reveal the translational potential of ultrasound to effectively modulate the PNS through intrinsic neuronal mechanisms.

The nervous system is a central command center that governs homeostasis in physiological and pathophysiological states. Virtually all tissues, including the skin, heart, lungs, and gut, and immune organs, such as the bone marrow, spleen, and lymph nodes, are innervated by neurons of the peripheral nervous system (PNS). These specialized neurons serve both afferent functions, sending sensory information to the brain, and efferent roles, delivering neural signals to organs to alter their physiological outputs (1). For example, in the case of injury or infection, PNS neurons represent an essential component of immune responses (2). The intersection between the PNS and effector organs thus represents an ideal target for therapeutic development. Indeed, peripheral neuromodulation devices are approved by the US Food and Drug Administration (FDA) or in clinical trials to treat wide-ranging diseases from depression to rheumatoid arthritis (3). These devices rely on implanted electrodes, which require surgical procedures that inherently carry risk (4, 5). Thus, noninvasive strategies to modulate PNS activity are an appealing alternative to treat chronic diseases.

Focused ultrasound (FUS) enables noninvasive neuromodulation of deep brain tissue and has shown promise as a therapeutic tool (6). More than 60 y ago, William Fry and colleagues demonstrated the reversible inhibitory effects of ultrasound on the central nervous system (CNS) of frogs, monkeys, and cats (7–9). Since that pioneering work, stimulation of the CNS with ultrasound has been shown to elicit action potentials in hippocampal slices, noninvasively stimulate intact motor circuits, and display therapeutic potential for seizure disruption in mammals (6, 10–13). Compared to the CNS, the effects of ultrasound stimulation on peripheral nerves are less clear. Ultrasound has been reported to both suppress and augment electrically evoked activity in the mammalian and invertebrate PNS (14–20). Notably, human psychophysical studies revealed that transdermal sonication induced somatic sensations such as touch, thermoreception, and pain, suggesting that ultrasound activates sensory neurons (15, 21–23). In addition, noninvasive sonication of the mouse sciatic nerve elicited muscle activity, indicating that FUS excites motor neurons (24, 25). Moreover, one report showed that sonication of a cat Pacinian corpuscle evoked neural activity consistent with receptor or action potentials (21). Despite these tantalizing studies, a systematic analysis of FUS-activated action potentials in mammalian peripheral neurons is lacking. This gap in knowledge is an impediment to the therapeutic development of PNS ultrasound neuromodulation, as protocols to reliably control neuronal activity have yet to be established despite decades of research efforts.

To address this gap, we sought to determine reliable FUS parameters that excite action potentials in mammalian peripheral neurons in intact tissue. We focused on mechanosensory neurons of mouse dorsal root ganglia, whose peripheral axons, or afferents, densely innervate skin and internal organs to convey sensory information to the CNS. Activation of primary sensory neurons gives rise to distinct sensations, including touch, pain, itch, warmth, and cold. These distinct percepts are initiated by an impressive array of somatosensory neuronal subtypes, including multiple classes of mechanoreceptors, thermoreceptors, and nociceptors (or pain-sensing neurons). Peripheral sensory neurons can be further classified based on neurophysiological properties, including conduction velocity (CV), receptive field (RF; the area of skin they innervate), sensory threshold, and firing pattern (26). Thus, these well-studied neurons provide a robust platform for examining the excitatory effects of FUS in intact mammalian tissue.

Here, we show that millisecond, high-intensity stimulation of sensory neurons with FUS is sufficient to elicit action potentials in all mechanosensory neurons studied. Moreover, the mechanically gated ion channel PIEZO2 sets the threshold for FUS activation of sensory neurons in peripheral tissues. These results define a parameter space to noninvasively excite sensory neurons in intact tissue and reveal molecular mechanisms that enable the transduction of sonication to neural activation—insights that have the potential to inform the development of neuromodulatory therapeutics.

## Results

### Ultrasound Evokes Action Potentials from Mouse Sensory Neurons.

**Fig. 1.**
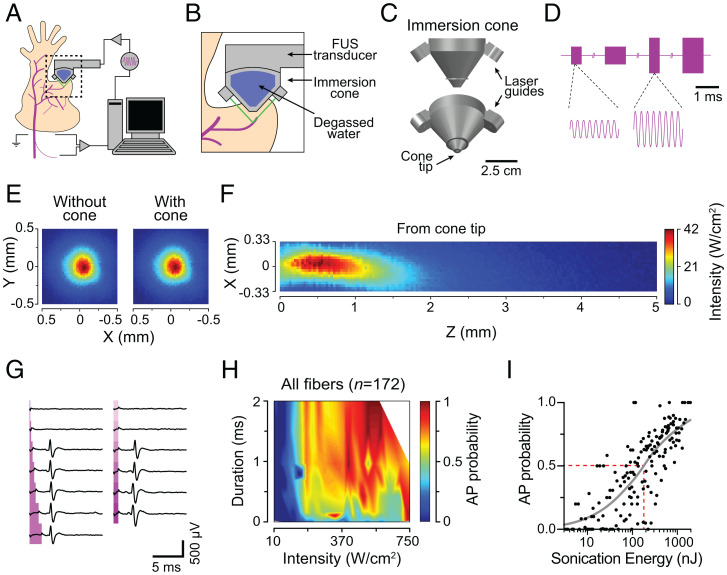
Ultrasound evokes action potentials from mouse sensory neurons. (*A*) Experimental setup. (*B*) Laser-guided (green) targeting of RFs. (*C*) Immersion cone. (*D*) Representative driving signal (3.57 MHz). Amplitude modulates intensity; cycle number modulates stimulus duration. (*E*) X-Y ultrasound beam profile without (*Left*) and with (*Right*) the cone demonstrates that the cone does not disrupt the beam. (*F*) X-Z beam profile measured from the cone tip (z = 0). (*G*) Representative FUS-evoked action potentials. Traces recorded sequentially from top to bottom. Magenta regions denote FUS stimulation. *Left*, Duration exploration (0.1, 0.25, 0.50, 1.0, 1.5, and 2.0 ms) with fixed intensity (155 W/cm^2^). *Right*, Intensity exploration (45 to 340 W/cm^2^, ∼55 W/cm^2^ steps) with fixed duration (0.75 ms). (*H*) Aggregate FUS parameter–probability space of all neurons recorded (*n* = 164 parameter sets; *Materials and Methods*). (*I*) Transform of *H* into total sonication energy. Gray line denotes fit to stimulus-response data (Max_AP_ = 1.0, slope = 0.79, EC_50_ = 186, E_50%Prob_ = 186, *R*^2^ = 0.64), where EC_50_ represents the half maximal effective energy relative to the fit maximum, and E_50%Prob_ represents the half maximal effective energy relative to a probability of 1.0. AP, action potential. Red dotted line denotes E_50%Prob_.

We developed an experimental paradigm using mouse ex vivo skin-nerve preparations that enabled simultaneous FUS stimulation and electrophysiological recordings from individual peripheral neurons (Fig. 1*A*). To accomplish targeted sonication of sensory neurons, we designed a custom, three-dimensional (3D)–printed immersion cone equipped with two lasers that intersected at the center of the ultrasound focus (Fig. 1 *B* and *C*). The immersion cone thus provided two technical advances: coupling of the ultrasound beam to the target tissue with degassed water, and laser-guided positioning of the FUS focus to tissues of interest. FUS was delivered using a transducer controlled by 3.57 MHz sinusoid waves delivered from a function generator and amplified with a radio-frequency amplifier (Fig. 1*D*). The resulting ultrasound beam in free-field had a focal diameter (full-width at half maximum) of 0.33 mm and a focal length of 1.16 mm from the cone tip (Fig. 1 *E* and *F*).

Given that acoustic waves produce primarily mechanical effects, we analyzed mechanosensory neurons that innervate skin and that initiate senses such as touch and mechanical pain (27). After establishing an extracellular recording from teased nerve fibers, a neuron’s RF was identified by gently pressing the skin with a blunt rod (∼5 mm diameter). Next, the RF was sonicated with laser-guided FUS. To identify efficient and reliable FUS protocols, neurons were sequentially stimulated with varying combinations of FUS parameters. Stimulus duration (0.1 to 2.0 ms in 0.1 to 0.5 ms steps) and intensity (11 to 743 W/cm^2^ in 25 to 60 W/cm^2^ steps) were varied, whereas ultrasound frequency (3.57 MHz) and interstimulus interval (5 s) remained fixed. Each FUS parameter set was presented 4 to 10 times, and action-potential probability was estimated as the fraction of stimuli that elicited an action potential. FUS stimulation within this range had negligible thermal effects (< 1 °C; FUS parameters: 2 ms, 743 W/cm^2^).

We explored > 100 FUS parameter combinations in mechanosensory neurons and found that high-intensity, millisecond sonication with FUS reliably excited single action potentials (Fig. 1*G*). Surprisingly, all recorded sensory neurons were excited by sonication (*n* = 172/172). Over the ranges tested, increasing either sonication duration or intensity sufficed to increase action-potential probability (Fig. 1*H*). Indeed, total sonication energy, which is proportional to the product of intensity and stimulus duration, showed a strong positive correlation with action-potential probability (Fig. 1*I*; *R* = 0.81, *P* < 0.0001; Spearman’s correlation). These data reveal an efficient FUS parameter space to excite peripheral neurons and indicate that the primary driver of FUS-evoked action potentials is the amount of energy delivered.

### Distinct Classes of Mechanosensory Neurons Are Excited by FUS.

**Fig. 2.**
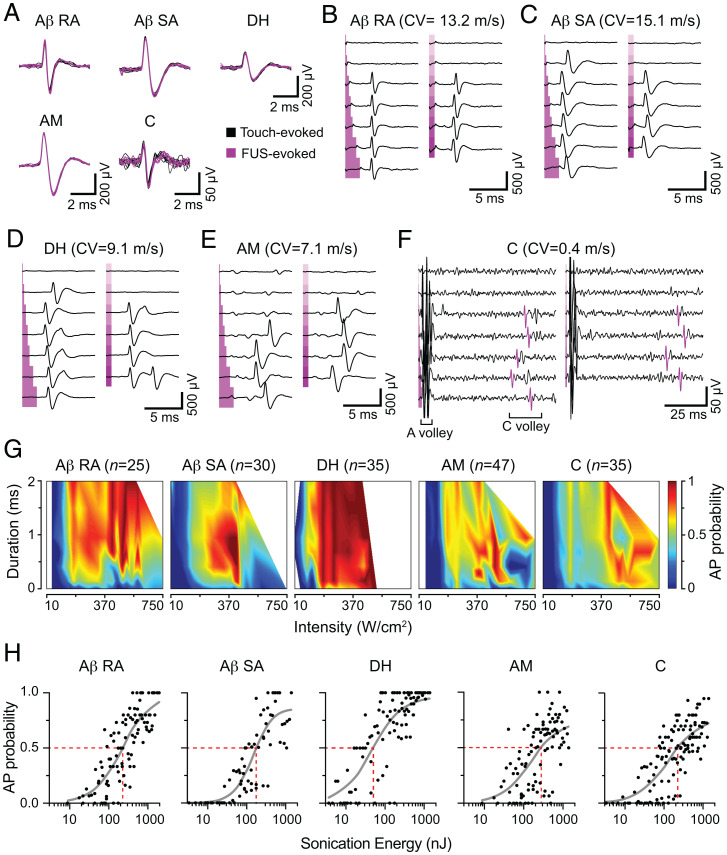
Distinct classes of mechanosensory neurons are excited by FUS. (*A*) Representative mechanically (black, seven traces) and ultrasound-evoked (magenta, seven traces) action potentials from afferents categorized by mechanosensory classes: Aβ RA, Aβ SA, DH, and AM. (*B*–*F*) Representative parameter space exploration from different afferent classes, displayed as in Fig. 1*G*. Each panel represents data from a single experiment. *Left*, Duration exploration (0.1, 0.25, 0.50, 1.0, 1.5, and 2.0 ms) at fixed intensity, as noted below. *Right*, Intensity exploration (unless noted, range = 45 to 340 W/cm^2^, ∼55 W/cm^2^ steps) with fixed duration (0.75 ms). (*B*) Aβ RA unit. Duration exploration intensity = 460 W/cm^2^. Intensity exploration range = 390 to 540 W/cm^2^, ∼30 W/cm^2^ steps. (*C*) Aβ SA unit. Duration exploration intensity = 155 W/cm^2^. (*D*) DH unit. Duration exploration intensity = 460 W/cm^2^. (*E*) AM unit. Duration exploration intensity = 460 W/cm^2^. (*F*) C-fiber unit. Duration exploration intensity = 220 W/cm^2^. Action potentials highlighted in purple display the spikes that were sorted and analyzed in the representative experiment. (*G*) Aggregate FUS parameter–probability space representation of all neurons recorded, separated by mechanosensory class. Pseudocolor axis represents action-potential probability (parameter sets: Aβ RA, *n* = 101; Aβ SA, *n* = 76; DH, *n* = 100; AM, *n* = 101; C-fiber, *n* = 125). (*H*) Transform of the data in *F* into total sonication energy (*Materials and Methods*). Data are displayed as the mean action-potential probability at each sonication energy sampled. Gray line denotes dose-response curve (*SI Appendix*, Table S2 for fit values). Red dotted line denotes E_50%Prob_.

Mechanosensory neurons that serve different roles in vivo can be functionally classified as ex vivo based on their electrophysiological properties. Aβ rapidly adapting (Aβ RA) and Aβ slowly adapting (Aβ SA) fibers are myelinated, fast-conducting fibers that encode tactile information. D-hair (DH) mechanoreceptors are intermediately conducting, Aδ fibers that report hair movement. Noxious mechanical stimuli are encoded by A-fiber mechanonociceptor (AM) and C-fibers, which have unmyelinated axons. Thus, we next asked whether these distinct classes of sensory neurons responded differentially to FUS parameter combinations by partitioning our neuronal dataset into these five classes (see *Materials and Methods* for classification): Aβ RA (*n* = 25), Aβ SA (*n* = 30), DH (*n* = 35), AM (*n* = 47), and C-fibers (*n* = 35; Fig. 2 *A*–*F* and *SI Appendix*, Table S1).

All neuronal classes examined were reliably excited by sonication. Comparison of the two-dimensional (2D) FUS parameter space by class revealed that short (∼0.75 ms), high-intensity (350 to 500 W/cm^2^) sonication was highly effective in evoking action potentials across all classes (Fig. 2*G*). To directly compare FUS sensitivity among classes, we analyzed total sonication energy, which positively correlated with action-potential probability in all fiber types (Fig. 2*H*; Aβ RA, *R* = 0.81; Aβ SA, *R* = 0.86; DH, *R* = 0.77; AM, *R* = 0.62; C, *R* = 0.76; *P* < 0.0001, Spearman’s correlation). For each fiber class, data were fit with a stimulus-response relation to estimate the maximal action-potential probability (Max_AP_) and the energy at which the probability of firing was 50% (E_50%prob_; *SI Appendix*, Table S2). Interestingly, DH neurons, which are ultrasensitive to light touch (28), were more likely to be excited by ultrasound than any other class (Max_AP_ = 0.97; E_50%prob_ = 50 nJ). By contrast, AM fibers, which have higher mechanical thresholds, were less excitable overall (Max_AP_ = 0.77; E_50%prob_ = 280 nJ). We also noted that Aβ and DH fibers had a higher Max_AP_ compared with AM and C-fibers (*SI Appendix*, Table S2); therefore, low-threshold mechanoreceptors follow FUS stimulation more reliably than nociceptors over this range. Together, these data define a range of FUS parameters (∼0.75 ms, 350 to 500 W/cm^2^, 350 to 550 nJ) capable of exciting all mechanosensory neurons.

We next asked whether sonication induced damage to target tissue. Skin was sonicated with a maximal FUS parameter set (1 ms, 743 W/cm^2^, 1301 nJ, 50 stimuli, 5 s interstimulus interval). Regions adjacent to and within the FUS focus were analyzed using hematoxylin and eosin stained cryosections (*SI Appendix*, Fig. S1 *A* and *B*). To assess for sonication-induced damage, we compared the thickness of the major skin compartments (epidermis, dermis + hypodermis). No significant differences were found (*SI Appendix*, Fig. S1*C*). Moreover, gross damage, such as tears, was not observed. These data suggest that the FUS parameters employed by this study do not induce substantial damage to targeted tissues.

**Fig. 3.**
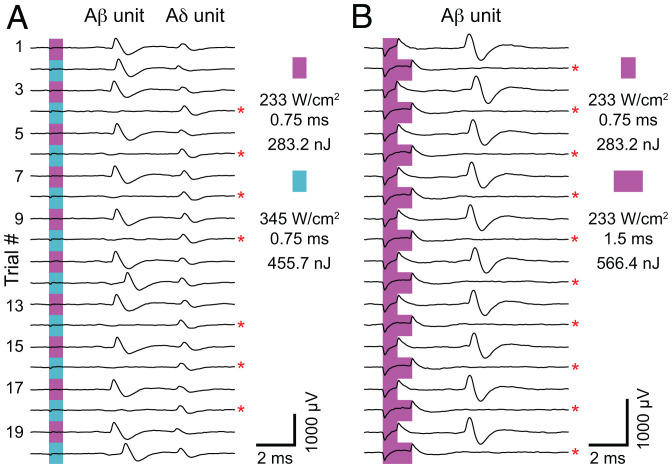
The probability of action-potential failure depends on sonication magnitude. (*A* and *B*) Representative experiments demonstrating action-potential failure (red asterisks) with increased FUS intensity (*A*) or increased FUS duration (*B*). Traces are plotted sequentially; the top trace (trial 1) was evoked first, and the traces below were recorded consecutively afterward (interstimulus interval, 5 s). In *A*, two units are excited by FUS (Aβ and Aδ). Note that increased sonication energy results in action-potential failure of the Aβ unit but not the Aδ unit.

Interestingly, some fibers displayed nonmonotonic tuning in their probability-response profiles. In these neurons, action-potential probability first increased and then decreased with progressively higher-energy FUS stimulation (high-intensity and/or long sonication duration). Indeed, in these neurons, alternating optimal FUS stimulation parameters with supraoptimal parameters enabled fiber-specific control of action-potential generation (Fig. 3). Notably, the failure to elicit action potentials with supraoptimal FUS stimulation did not represent damage, as optimal stimulation consistently elicited action potentials within 5 s of supraoptimal stimulation. Only a fraction of total neurons with supraoptimal FUS stimulation displayed this type of response to high-energy FUS (high-intensity, *n* = 20/164; long-duration, *n* = 22/136), suggesting that intrinsic properties of neural subsets are important for this tuning phenomenon.

### Nerve Trunk FUS Stimulation Excites Action Potentials.

**Fig. 4.**
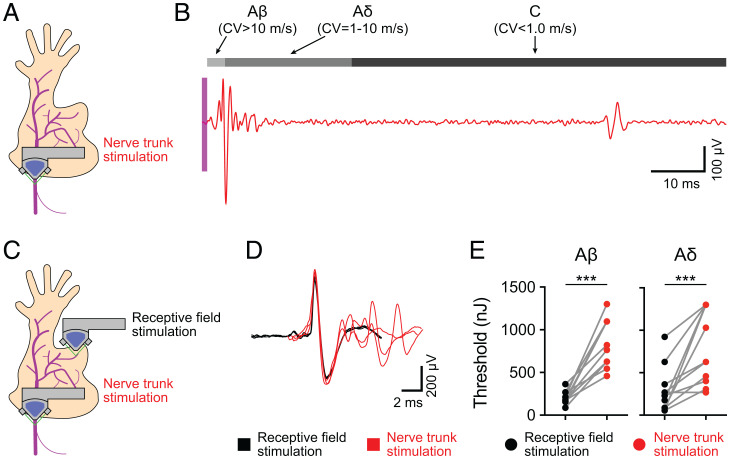
NT FUS stimulation evokes action potentials. (*A*) Overview of experimental setup. NTs were targeted with FUS, and action potentials were recorded. (*B*) Representative recording, demonstrating FUS-evoked (magenta region denotes time of FUS stimulation) compound action potentials from Aβ, Aδ, and C-fibers. (*C*) Experimental setup to compare RF and NT FUS stimulation. Mechanosensory afferents were first identified with the manual exploration of RFs with a blunt glass probe. Once identified and classified, afferents were sequentially stimulated with FUS targeted to NTs and RFs. (*D*) Comparison of FUS-evoked action potentials elicited by RF stimulation (black) and NT stimulation (red). (*E*) Comparison of sonication energy thresholds (> 50% probability of eliciting an action potential) from FUS stimulation targeted to RFs or NTs from the same fiber. Gray lines represent thresholds from the same fiber. *Left*, Aβ fibers; *Right*, Aδ fibers (***P = 0.001; Wilcoxon matched-pairs signed rank test; *n* = 11 in Aβ fibers and *n* = 12 in Aδ fibers).

One therapeutic application of FUS neuromodulation is the noninvasive stimulation of nerves, such as the vagus nerve, to manipulate neurohumoral reflexes. Such a device would require stimulation of the nerve trunks (NTs) rather than the RFs. Thus, we tested whether FUS sonication of peripheral NTs evokes action potentials. To do so, we stimulated the saphenous NT with FUS, which elicited compound action potentials composed of Aβ, Aδ, and C-fiber activity (Fig. 4 *A* and *B*). We next compared FUS stimulation of RFs versus NTs for individual mechanosensory neurons (Fig. 4*C*). Stimulation of the NTs activated the same action-potential waveform generated by RF stimulation (Fig. 4*D*). Notably, NT stimulation activated several units in addition to the target neuron that were likely spatially collocated within the peripheral nerve. Interestingly, we found that the 50% threshold to activate action potentials in NTs was significantly higher than those observed for RFs in both Aβ (medians: RF, 175 nJ; NT, 764 nJ) and Aδ fibers (medians: RF, 203 nJ; NT, 627 nJ; Fig. 4*E*). These findings demonstrate that neurons can be targeted either at RFs or along NTs and define effective sonication ranges for activation in both stimulus paradigms.

### FUS Evokes Action Potentials with Millisecond Latencies Compared with Electrical Stimulation.

**Fig. 5.**
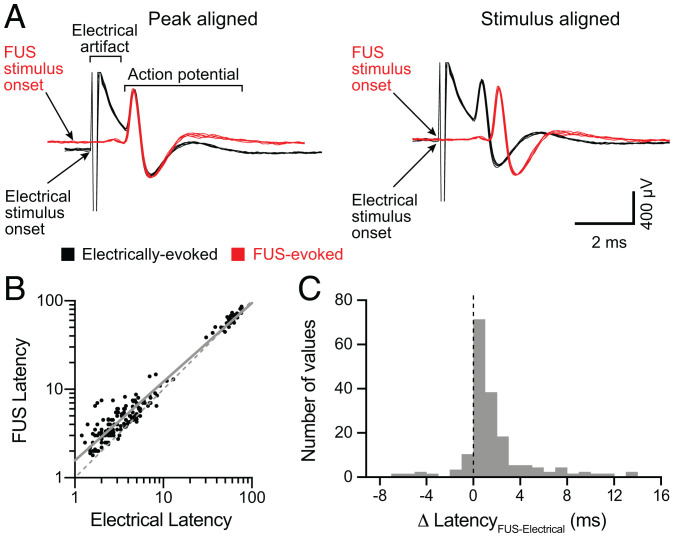
FUS evokes action potentials with millisecond latencies compared with electrical stimulation. (*A*) Comparison of representative FUS-evoked (red) to electrically evoked (black) action potentials from the same Aβ SA fiber (CV = 15.0 m/s). *Left*, Action potentials aligned to peak. *Right*, Action potentials aligned to the onset of stimulation (arrows indicate stimulus onset). (*B*) Scatterplot of log-transformed FUS-evoked versus electrically evoked action-potential latencies (defined as time from stimulus onset to action-potential positive peak). Gray solid line denotes linear regression of log-transformed data (slope = 0.89; y-intercept = 0.20, *R*^2^ = 0.95). Gray dotted line denotes y = x. (*C*) Histogram of the difference between FUS-evoked and electrically evoked action-potential latencies from each neuron.

A number of FDA-approved neuromodulation devices employ electrical nerve stimulation (3). These technologies directly depolarize neurons to activate voltage-gated sodium channels, which faithfully and rapidly trigger action potentials. Given that our data reveal specific FUS parameters that reliably activate one-to-one action potentials, we wondered how FUS stimulation compares to electrical stimulation in terms of speed. Analysis of peak-aligned waveforms for individual neural responses showed that electrically evoked spike waveforms closely resembled those elicited by FUS for all fibers examined. These data indicated that the same fibers are activated by both stimuli (Fig. 5*A*, *Left*). When waveforms were aligned by stimulus onset, FUS-evoked action-potential latencies were ∼1 ms longer than those measured from electrical stimulation (Fig. 5*A*, *Right*). Latencies for FUS- and electrically evoked action potentials were positively correlated (A-fibers, *R* = 0.80, *P* < 0.0001; C-fibers, *R* = 0.85, *P* < 0.0001; Spearman’s correlation) and were well fit by linear regression with a positive intercept and a shift toward the FUS axis (Fig. 5*B*; slope, 0.89 ms; *y*-intercept, 0.20 ms; *R*^2^ = 0.95). Indeed, across the population, the difference between FUS and electrical latencies (Δ Latency) measured from the same fiber was in the millisecond range (median = 0.9 ms, interquartile range = 1.8 ms, *n* = 172; Fig. 5*C*). These data indicate that the molecular mechanisms that underlie FUS-evoked stimulation of peripheral neurons activate within milliseconds, which is consistent with neuronal ion channels.

### FUS Stimulates Action Potentials in Part through the Mechanically Activated Ion Channel PIEZO2.

**Fig. 6.**
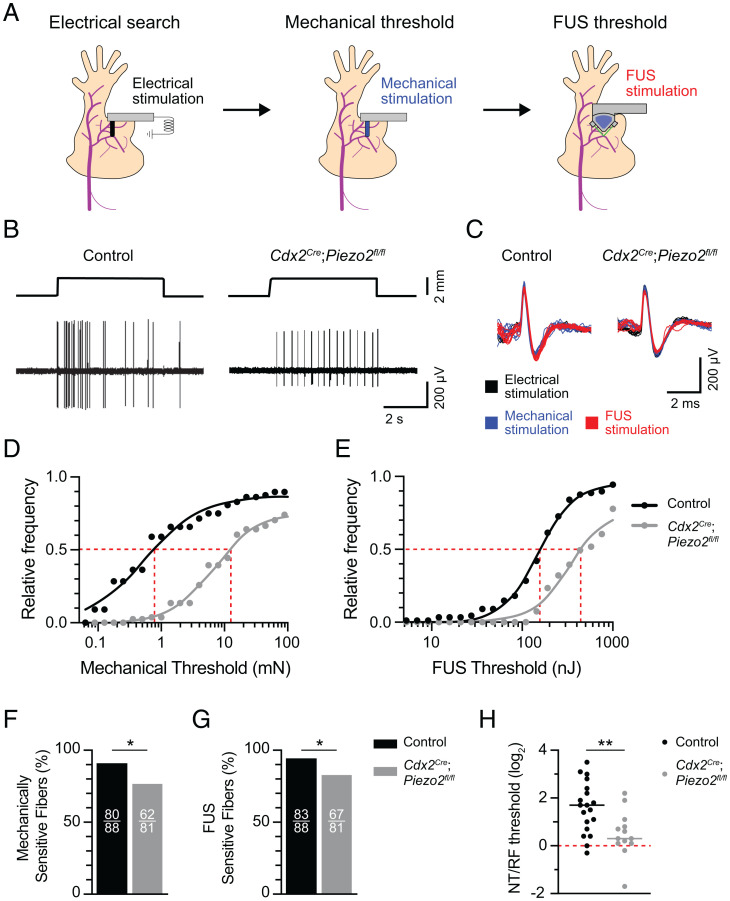
FUS stimulates action potentials at sensory RFs in part through the mechanically activated ion channel PIEZO2. (*A*) Experimental overview. Afferents were identified using an electrical search (*Materials and Methods*). Next, electrically identified RFs were stimulated with mechanical and FUS stimulation to measure mechanical and sonication energy thresholds, respectively. (*B*) Representative mechanically evoked responses from control (*Left*) and *Cdx2^Cre^*;*Piezo2^fl/fl^* (*Right*) mice. *Top* traces, displacement; *Bottom* traces, action-potential trains. (*C*) Representative action-potential traces evoked by electrical (black), mechanical (blue), and FUS (red) stimuli from control and *Cdx2^Cre^*;*Piezo2^fl/fl^* mice. (*D* and *E*) Cumulative response plots for mechanical (*D*) and FUS thresholds (*E*) from control and *Cdx2^Cre^*;*Piezo2^fl/fl^* mice (control: *n* = 88 fibers, five mice; *Cdx2^Cre^*;*Piezo2^fl/fl^: n* = 81 units, six mice). Black and gray lines denote stimulus-response fits (*SI Appendix*, Table S3 for fit values). Gray dotted line denotes E_50%Respond_. (*F* and *G*) Proportions of mechanically (*F*) and FUS-sensitive (*G*) fibers in control (black) and *Cdx2^Cre^*;*Piezo2^fl/fl^* (gray) mice. Numbers of sensitive fibers are listed as insets within bars. In *F*, *P = 0.01; in *G*, *P = 0.02 (two-sided Fisher’s exact test). (*H*) FUS thresholds at NTs and RFs were measured for individual A-fiber units (control: *n* = 19 fibers, three mice; *Cdx2^Cre^*;*Piezo2^fl/fl^: n* = 13 fibers, four mice). NT/RF threshold ratios were calculated and log_2_ transformed to achieve normally distributed populations. Means were compared with unpaired, two-tailed Student’s *t* test (**P = 0.006).

FUS may excite action potentials either by directly activating voltage-gated sodium channels or by activating upstream sensory ion channels that depolarize neurons to the action-potential threshold. Given that FUS-evoked action-potential latencies are consistently ∼1 ms longer than electrical stimulation, we hypothesized that FUS activates fast sensory ion channels, such as mechanically gated ion channels. Mechanically gated ion channels encoded by *Piezo2* constitute the principal mechanotransduction mechanism in mammalian A-fiber mechanosensory neurons (29, 30). Thus, we next asked whether activation of peripheral neurons with sonication requires PIEZO2. We generated *Cdx2^Cre^*;*Piezo2^fl/fl^* mice, which harbor a deletion of *Piezo2* in caudal tissues including peripheral neurons (29, 31, 32). Mechanosensitivity is significantly reduced in A-fiber mechanosensory neurons lacking functional PIEZO2 (29, 30); thus, an electrical search method was used to identify A-fiber responses (both Aβ and Aδ fibers) from *Cdx2^Cre^*;*Piezo2^fl/fl^* and control genotypes (33). Electrically identified receptive fields were then sequentially targeted with mechanical and FUS stimulation to measure action-potential thresholds (Fig. 6 *A*–*C*). To compare these thresholds across genotypes, we analyzed cumulative response profiles of A-fibers from each stimulus. Data were then fit with stimulus-response relations to estimate the stimulus magnitude at which the 50% of fibers responded (E_50%Respond_; *SI Appendix*, Table S3). Peripheral neurons from *Cdx2^Cre^*;*Piezo2^fl/fl^* mice displayed a marked increase in mechanical thresholds (E_50%Respond_ = 11.5 mN) compared with control genotypes (E_50%Respond_ = 0.8 mN; Fig. 6*D*). Indeed, few fibers showed mechanical thresholds in the innocuous range (≤ 4 mN), confirming published reports that PIEZO2 channels are essential for neural responses to touch stimuli (29). Likewise, afferents from *Cdx2^Cre^*;*Piezo2^fl/fl^* mice were substantially less sensitive to FUS sonication (E_50%Respond_ = 447.5 nJ) compared with controls (E_50%Respond_ = 158.5 nJ; Fig. 6*E*). We also observed fewer mechanically and FUS-sensitive fibers in *Cdx2^Cre^*;*Piezo2^fl/fl^* mice compared with control genotypes (Fig. 6 *F* and *G*). Together, these data demonstrate that PIEZO2 is essential for mechanically and FUS-evoked firing in a subset of sensory neurons and suggest that this ion channel lowers the threshold for FUS stimulation in sensory neurons overall.

The decreased FUS sensitivity of PIEZO2 knockout mice is reminiscent of the differences we observed between NT and RF stimulation (Fig. 4*E*). Thus, we wondered whether PIEZO2 can account for elevated FUS threshold observed in nerve bundles compared with RFs. To answer this question, we performed FUS sonication of RFs and NTs for individual neurons from *Cdx2^Cre^*;*Piezo2^fl/fl^* mice and control littermates (Fig. 6*H*). As in our previous experiment, FUS thresholds of NTs were substantially higher than those of RFs (NT/RF threshold ratio = 3.9 ± 2.9, mean ± SD). By contrast, the NT/RF threshold ratio in *Cdx2^Cre^*;*Piezo2^fl/fl^* mice was 1.8 ± 1.2 (mean ± SD). Together, these data identify PIEZO2 as an intrinsic molecular effector of FUS neuromodulation in peripheral tissues and demonstrate that PIEZO2-independent mechanisms mediate neuromodulation in NTs.

## Discussion

Technologies that confer targeted, noninvasive modulation of the nervous system have been long sought in translational neuroscience. Recent studies have revealed the therapeutic potential of noninvasive neuromodulation with ultrasound (6, 10, 11, 34, 35). Much of the progress in understanding the effects of ultrasound on neuronal tissue has been limited to the CNS. Our study demonstrates that ultrasound sonication directly and robustly evokes action potentials from individual neurons in the mammalian PNS. We show that millisecond, high-intensity (350 to 500 W/cm^2^) sonication of neuronal RFs is sufficient to elicit action potentials in both myelinated (Aβ and Aδ) and unmyelinated (C) fibers. Notably, action potentials follow FUS sonication in a one-to-one manner, demonstrating that FUS has the potential to allow tight temporal control over neuronal activity in the PNS. FUS stimulation of NTs excites action potentials effectively, although at higher sonication energies. Interestingly, the threshold of FUS activation at RFs is set by the mechanically activated ion channel PIEZO2. These findings reveal effective parameters for the noninvasive excitation of peripheral nerves with ultrasound in intact tissue, satisfying a critical step toward the development of ultrasound-based therapeutics.

Several mechanisms have been proposed to explain excitatory neuromodulation with ultrasound, including activation downstream of the thermal and mechanical effects of sonication (36–38). In our study, we did not observe a significant increase in temperature with maximal FUS stimulus parameters (*SI Appendix*, Fig. S2). These data agree with previous studies that report that ultrasound stimulation occurs under conditions that minimally heat tissues (10, 11, 39). Together, this information indicates that the thermal effects of sonication under our experimental conditions are minimal and do not explain the robust and repeatable neuronal activation that we observed.

A second possibility is that sonication induces mechanical effects on neural tissue, such as radiation force, membrane oscillation, or cavitation, resulting in ion-channel activation and action-potential generation. Indeed, radiation force has been shown to activate mechanosensitive MEC-4 channels in *Caenorhabditis elegans* (40). Our data support this model, as the deletion of the mechanically activated ion channel PIEZO2 disrupts the FUS-induced activation of peripheral neurons (Fig. 6*E*). Consistent with this role for PIEZO2, we observed that some low-threshold mechanoreceptors were more sensitive to FUS stimulation than nociceptors (Fig. 2*H*). Previous studies of the effects of sonication on PIEZO channels have yielded inconsistent results. One report in dental stem cells indicates that sonication-induced cell proliferation is mediated by PIEZO proteins; however, the researchers were not able to distinguish between PIEZO1 and PIEZO2 (41). Two groups have reported that heterologously expressed PIEZO1 in CHO and HEK cells is activated by sonication in vitro. Both studies propose a mechanism that relies on acoustic fluid streaming, which is less likely to occur in vivo (42, 43). PIEZO1 has been shown to mediate calcium responses in in vitro cortical neurons in two independent studies (44, 45). Interestingly, a technique for the noninvasive activation of a chimeric antigen receptor expressing T cells with FUS is proposed to be mediated through PIEZO1, which may have translational potential for oncological therapeutics (46).

Our data demonstrate that PIEZO2 confers efficient sonication-induced activation of peripheral neurons at their RFs, the location where they innervate peripheral tissues. We note, however, that the genetic deletion of PIEZO2 abolished action potentials evoked by either FUS or mechanical stimuli in only a subset of neurons. Moreover, NT activation is less sensitive to FUS than RF stimulation, and this activity is independent of PIEZO2. These results suggest that other mechanisms can transduce sonication into neural activity (Fig. 6*D*). For example, OSCA/TMEM63 proteins, which are an evolutionarily conserved family of mechanosensitive ion channels, may be activated by sonication (47). In hippocampal slices, ultrasound has been proposed to stimulate action potentials through the mechanical activation of voltage-gated sodium and calcium channels (10). A recent in vitro study in *Xenopus* oocytes demonstrated that FUS directly activates TRAAK channels, which are mechanosensitive potassium channels (48, 49). The activation of potassium channels, which results in decreased neuronal excitability, cannot account for action-potential initiation but may explain our observation that the probability of FUS-evoked action potentials decreases at higher FUS stimulus intensities. Future studies are needed to reveal the complete set of ion channels that underlie the transduction of sonication to neural activity.

The use of ultrasound to activate mammalian peripheral neurons was first demonstrated more than 40 y ago (21, 50). A handful of studies have shown that ultrasound sonication to human skin initiates somatic sensations such as warmth, pain, and pressure (22, 50); however, the potential therapeutic applications of the neuromodulation of peripheral nerve activity extend beyond sensory modulation. One such application is the noninvasive modulation of the neural reflex arc to treat chronic disease (5). The neural reflex arc is composed of peripheral afferent neurons that signal to the CNS and efferent neurons that send regulatory signals to virtually all peripheral tissues (51). Stimulation of the vagus nerve, which is composed of both afferent and efferent neurons, is an FDA-approved intervention for epilepsy and treatment-resistant depression, and it has shown promise for diseases such as rheumatoid arthritis, systemic lupus erythematosus, Crohn’s disease, and hypertension (52). A limitation of current approved therapeutics for vagus nerve stimulation is that they rely on surgically implanted electrodes, which can result in significant complications (53). Our data support the feasibility of developing a noninvasive, ultrasound-based device that could act as a substitute for the surgical implantation of electrodes in vagus nerve–targeting therapeutics. Notably, we found that the axons of peripheral neurons within NTs were reliably excited by FUS stimulation.

We were intrigued to find that neuronal subtypes had differential sensitivities to FUS stimulation. Indeed, DH neurons, which are highly sensitive, low-threshold mechanoreceptors that innervate hair follicles, were the neurons most sensitive to FUS stimulation. By contrast, AM neurons, which are high-threshold mechanoreceptors that trigger pain, required greater sonication energies to activate. Moreover, a handful of neurons displayed nonmonotonic dose-response relationships to FUS stimulation and were suppressed at larger stimulation magnitudes, mirroring a recent study in mouse motor neurons (54). Although our study did not identify unique FUS parameter sets that selectively activate functionally distinct subsets of neurons, our results provide a foundation for future studies that aim to do so. Selective activation would amplify the therapeutic potential of FUS neuromodulation, as one might be able to target defined neuronal subsets within mixed nerves. The vagus nerve, for example, contains afferent and efferent neurons that innervate most visceral tissues, including the heart, lungs, gut, and immune organs. Pathologies of specific organs, or organ systems, may benefit from noninvasive and selective neuromodulation of specific subsets of vagal neurons.

## Materials and Methods

### Ethical Approval.

Animal use was conducted according to guidelines from the NIH Guide for the Care and Use of Laboratory Animals and was approved by both the Institutional Animal Care and Use Committee of Columbia University Medical Center and the Animal Care and Use Review office of the United States Army Medical Research and Materiel Command.

### Animals.

Mice were maintained on a 12 h light/dark cycle, and food and water were provided ad libitum. Euthanasia was performed with isoflurane inhalation followed by cervical dislocation, as approved by institutional guidelines (55). Experiments were performed on 7- to 13-wk-old mice. The following strains were used in this study: female C57BL/6 (Jackson Labs), *Cdx2^Cre^* (31), and *Piezo2^fl/fl^* (56). Details on tissue recombination for *Cdx2^Cre^* mice are published elsewhere (57). For experiments involving the tissue-specific deletion of *Piezo2*, genotypes that lacked either Cre or floxed *Piezo2* alleles (*Piezo2^fl/fl^* or *Piezo2^fl/+^*) were designated as littermate controls, and *Cdx2^Cre^*;*Piezo2^fl/fl^* were experimental animals (32). Automated genotyping was performed through Transnetyx (http://www.transnetyx.com/).

### Ultrasound Stimulation.

FUS was delivered with a commercial focused ultrasound transducer with a 3.57 MHz center frequency (35 mm focal depth; SU-107, Sonic Concepts). Driving signals were delivered by a function generator (33220A; Keysight Technologies) and amplified through a 150 W amplifier (A150; Electronics & Innovation). To calibrate the transducer (*SI Appendix*, Table S4), beam plots were acquired using a fiber-optic hydrophone (HFO690; Onda). The transducer was mounted on a 3D motorized XYZ positioner (Bislide; Velmex). After locating the center of the ultrasound focus, 2D raster scans in both the XY and XZ planes were acquired (100 cycle bursts and a 10 Hz pulse repetition frequency).

To deliver targeted FUS stimulation of neurons, we constructed a custom immersion cone, equipped with guide lasers (VLM-650-01 LPA; Quarton USA) to identify the ultrasound focus. The cone was filled with degassed water and the tip was sealed with a thin plastic membrane (CE0434; EMT Medical Co.). Using the intersection of the lasers as a guide, the focus of the transducer was positioned with a 3D micromanipulator (MPC-200; Sutter Instrument) directly on the RF or the saphenous NT. To ensure the continuous coupling of the transducer to the target, a small volume of bath solution was maintained between the tip of the immersion cone and the target surface.

The FUS parameters employed were stimulus duration (0.1 to 2.0 ms, 0.1 to 0.5 ms steps), intensity (11 to 743 W/cm^2^, 25 to 60 W/cm^2^ steps), ultrasound frequency (3.57 MHz), and interstimulus interval (5 s). The stimulus order was typically from short to long duration and low to high intensity. The latency of FUS-evoked action potentials was measured from the FUS trigger to the action-potential peak.

RFs or NTs were stimulated >4 times, with an interstimulus interval of 5 s. FUS thresholds were defined as the first sonication energy that generated action potentials in >50% of stimulus presentations.

Temperature measurements were obtained with a thermistor (TA-29; Warner Instruments) placed <1 mm from the FUS focus (to avoid artifact). Ultrasound was delivered sequentially (stimulus duration 2.0 ms; intensity, 40 to 743 W/cm^2^, 25 to 60 W/cm^2^ steps; ultrasound frequency, 3.57 MHz; interstimulus interval, 5.0 s), and instantaneous changes in temperature were measured with a temperature controller (TC-344B; Warner Instruments) sampled at 20 kHz.

### Ex Vivo Skin-Nerve Electrophysiology.

Action potentials from teased nerve fibers were recorded after dissecting the mouse hindlimb skin and saphenous nerve according to published methods (58). Tissue was placed with the epidermis side up in a custom chamber and perfused with carbogen-buffered synthetic interstitial fluid (in mM: 108 NaCl, 3.5 KCl, 0.7 MgSO_4_, 26 NaHCO_3_, 1.7 NaH_2_PO_4_, 9.5 sodium gluconate, 5.5 glucose, 7.5 sucrose, and 1.5 CaCl_2_, saturated with 95% O_2_-5% CO_2_; pH 7.4) kept at 32 °C with a temperature controller (TC-344B; Warner Instruments). The nerve was kept in mineral oil in a recording chamber, teased, and placed onto a recording electrode connected with a reference electrode to a differential amplifier (model 1800; A-M Systems). The extracellular signal was digitized using a PowerLab 8/35 board (AD Instruments) and recorded using LabChart software (AD Instruments). Sampling frequencies were 20 kHz or 40 kHz.

Single units and their RFs were identified using mechanical search with a blunt glass probe. Once isolated, afferents were characterized based on mechanical threshold, RF characteristics, CV, and adaptation properties to sustained mechanical stimuli. The mechanical threshold was measured by stimulating RFs with calibrated von Frey monofilaments and was defined as the first von Frey monofilament that generated action potentials in >50% of stimulus presentations. RFs and responses to hair movement were evaluated under stereomicroscopy by deflecting individual hairs with fine forceps (Model SZX16; Olympus). CV was estimated based on the electrical stimulation of RFs delivered from a pulse stimulator (Model 2100; A-M Systems) and calculated as the quotient of distance between the stimulus and recoding electrodes and the latency of the action-potential peak from the stimulus artifact. To assess adaptation properties, RFs were stimulated with a custom-built, computer-controlled mechanical stimulator (tip diameter, 1.6 mm).

For experiments in *Cdx2^Cre^*;*Piezo2^fl/fl^* and littermate control mice, an electrical search was used to identify afferents (33). To do so, electrical stimulation was delivered first near where the saphenous nerve inserts into the skin and progressively more distal to approximate the RF locations. Once electrically identified RF locations were established, mechanical thresholds, RF characteristics, CV, and adaptation properties to sustained mechanical stimuli were estimated as described above.

### Sensory Afferent Classification.

Mechanosensory afferents were classified into five subtypes based on physiological response properties (*SI Appendix*, Table S1): Aβ RA, Aβ SA, DH, AM, and C-fibers. Classification was performed based on criteria modified from previously published work (26): Aβ RA fibers, CV >∼10 m/s, no response to zigzag hair movement, RA responses to 5 s mechanical stimulation; Aβ SA fibers, CV >∼10 m/s, response to touch-dome indentation and/or hair movement, sustained responses to 5 s mechanical stimulation; DH fibers, CV ≥ 1 m/s and ≤ 10 m/s, responses to zigzag hair movement; AM fibers, CV ≥ 1 m/s and ≤ 10 m/s, no response to hair movement, and SA responses to 5 s mechanical stimulation; C-fibers, CV < 1 m/s.

### Data Analysis.

Spike sorting and data analysis was performed in Matlab. Spikes were sorted based on the following parameters: positive peak amplitude, negative peak amplitude, positive peak rise time, spike width, and negative peak decay time. Sorted waveforms were then averaged to generate a template, which was then compared back to the sorted waveforms with correlation analysis. Spikes kept for further analysis had correlation coefficients of >0.97 in A-fibers and >0.85 in C-fibers.

Action-potential probability was calculated for each FUS parameter combination delivered to each recorded neuron. To generate aggregate parameter exploration data (Figs. 1 and 2), action-potential probabilities for each sampled FUS parameter combination were averaged across fibers. Given that the parameters delivered to each recorded fiber varied, only parameters that were delivered to at least two fibers were considered for further analysis. To generate continuous surface plots, probability data were interpolated with the “scatteredInterpolant” function using linear interpolation. Sonication energy was calculated from aggregate FUS parameter–probability datasets using the following function:

$$\text{Sonication}\;\text{Energy}\;(J)=I\times \pi r^{2}\times t.$$

$$I=\text{intensity}\;\left(W/{\text{cm}}^{2}\right);r=\text{US}\;\text{focal}\;\text{radius}\;\left(\text{cm}\right);t=\text{sonication}\;\text{duration}\;(\text{s}).$$

Sonication energy (Figs. 1*I* and 2*H*) and cumulative response profiles (Fig. 6 *D* and *E*) were fit with the following dose-response function:

$$y=\frac{a}{1+10^{\left(\left(\left({\log\;}_{10}b\right)\;-\;x\right)\;\times \;c\right)}};\;a=\text{Top};\;b=EC_{50};\;c=\text{slope}.$$

### Statistical Analysis.

Statistical analysis was performed in Matlab (R2018b; MathWorks) and Prism (GraphPad). Statistical parameters are described in the figure legends. A paired or unpaired Student’s two-tailed *t* test was used to compare means of two normally distributed, paired or unpaired groups, respectively. Log-normal populations were log-transformed to achieve normality before comparing means. The Wilcoxon signed rank test was used to compare the medians of two nonparametric groups. Paired one-way ANOVA was used to compare three or more normally distributed, paired groups. Nonparametric data with three or more groups were analyzed using the Kruskal–Wallis test. Correlations between nonparametric groups was computed using Spearman’s rank-order correlation. The normality of population data was assessed using the Kolmogorov–Smirnov test with Dallal–Wilkinson–Lilliefors *P* values, with *P* < 0.05 indicating nonnormality. Differences were considered significant if *P* < 0.05. All significance tests were justified considering the experimental design.

## Supplementary Material

Supplementary File

## Data Availability

All data used for the analyses described in this manuscript are freely available and have been deposited in an online repository (http://www.github.com/buh2003/FUSPiezo2) (59).

## References

[1] B. U. Hoffman, “The peripheral nervous system: From molecular mechanisms to non-invasive therapeutics,” PhD thesis, Columbia University, New York, NY (2019).

[2] L. Steinman, Elaborate interactions between the immune and nervous systems. Nat. Immunol. 5, 575–581 (2004). 15164017 10.1038/ni1078

[3] R. L. Johnson, C. G. Wilson, A review of vagus nerve stimulation as a therapeutic intervention. J. Inflamm. Res. 11, 203–213 (2018). 29844694 10.2147/JIR.S163248 PMC5961632

[4] K. Famm, B. Litt, K. J. Tracey, E. S. Boyden, M. Slaoui, Drug discovery: A jump-start for electroceuticals. Nature 496, 159–161 (2013). 23579662 10.1038/496159a PMC4179459

[5] K. Birmingham , Bioelectronic medicines: A research roadmap. Nat. Rev. Drug Discov. 13, 399–400 (2014). 24875080 10.1038/nrd4351

[6] Y. Tufail, A. Yoshihiro, S. Pati, M. M. Li, W. J. Tyler, Ultrasonic neuromodulation by brain stimulation with transcranial ultrasound. Nat. Protoc. 6, 1453–1470 (2011). 21886108 10.1038/nprot.2011.371

[7] W. J. Fry, Intense ultrasound; A new tool for neurological research. J. Ment. Sci. 100, 85–96 (1954). 13152523 10.1192/bjp.100.418.85

[8] F. J. Fry, H. W. Ades, W. J. Fry, Production of reversible changes in the central nervous system by ultrasound. Science 127, 83–84 (1958). 13495483 10.1126/science.127.3289.83

[9] W. J. Fry, V. J. Wulff, D. Tucker, F. J. Fry, Physical factors involved in ultrasonically induced changes in living systems: I. Identification of non‐temperature effects. J. Acoust. Soc. Am. 22, 867–876 (1950).

[10] W. J. Tyler , Remote excitation of neuronal circuits using low-intensity, low-frequency ultrasound. PLoS One 3, e3511 (2008). 18958151 10.1371/journal.pone.0003511 PMC2568804

[11] Y. Tufail , Transcranial pulsed ultrasound stimulates intact brain circuits. Neuron 66, 681–694 (2010). 20547127 10.1016/j.neuron.2010.05.008

[12] J. S. Manlapaz, K. E. Astroem, H. T. Ballantine Jr., P. P. Lele, Effects of ultrasonic radiation in experimental focal epilepsy in the cat. Exp. Neurol. 10, 345–356 (1964). 14211931 10.1016/0014-4886(64)90005-6

[13] B. K. Min , Focused ultrasound-mediated suppression of chemically-induced acute epileptic EEG activity. BMC Neurosci. 12, 23 (2011). 21375781 10.1186/1471-2202-12-23 PMC3061951

[14] R. R. Young, E. Henneman, Functional effects of focused ultrasound on mammalian nerves. Science 134, 1521–1522 (1961). 14009369 10.1126/science.134.3489.1521

[15] T. C. Dickey , Intense focused ultrasound can reliably induce sensations in human test subjects in a manner correlated with the density of their mechanoreceptors. Ultrasound Med. Biol. 38, 85–90 (2012). 22104527 10.1016/j.ultrasmedbio.2011.09.020 PMC3245865

[16] P. P. Lele, Effects of focused ultrasonic radiation on peripheral nerve, with observations on local heating. Exp. Neurol. 8, 47–83 (1963).

[17] P. H. Tsui, S. H. Wang, C. C. Huang, In vitro effects of ultrasound with different energies on the conduction properties of neural tissue. Ultrasonics 43, 560–565 (2005). 15950031 10.1016/j.ultras.2004.12.003

[18] R. T. Mihran, F. S. Barnes, H. Wachtel, Temporally-specific modification of myelinated axon excitability in vitro following a single ultrasound pulse. Ultrasound Med. Biol. 16, 297–309 (1990). 2363236 10.1016/0301-5629(90)90008-z

[19] E. J. Juan, R. González, G. Albors, M. P. Ward, P. Irazoqui, Vagus nerve modulation using focused pulsed ultrasound: Potential applications and preliminary observations in a rat. Int. J. Imaging Syst. Technol. 24, 67–71 (2014). 25165410 10.1002/ima.22080 PMC4142523

[20] C. J. Wright, S. R. Haqshenas, J. Rothwell, N. Saffari, Unmyelinated peripheral nerves can be stimulated in vitro using pulsed ultrasound. Ultrasound Med. Biol. 43, 2269–2283 (2017). 28716433 10.1016/j.ultrasmedbio.2017.05.008

[21] L. R. Gavrilov , The effect of focused ultrasound on the skin and deep nerve structures of man and animal. Prog. Brain Res. 43, 279–292 (1976). 1257484 10.1016/S0079-6123(08)64360-5

[22] W. Legon, A. Rowlands, A. Opitz, T. F. Sato, W. J. Tyler, Pulsed ultrasound differentially stimulates somatosensory circuits in humans as indicated by EEG and FMRI. PLoS One 7, e51177 (2012). 23226567 10.1371/journal.pone.0051177 PMC3514181

[23] T. Riis, J. Kubanek, Effective ultrasonic stimulation in human peripheral nervous system. IEEE Trans Biomed Eng. 69, 15–22 (2021). 34057888 10.1109/TBME.2021.3085170 PMC9080060

[24] M. E. Downs , Non-invasive peripheral nerve stimulation via focused ultrasound in vivo. Phys. Med. Biol. 63, 035011 (2018). 29214985 10.1088/1361-6560/aa9fc2

[25] S. A. Lee, H. A. S. Kamimura, M. T. Burgess, E. E. Konofagou, Displacement imaging for focused ultrasound peripheral nerve neuromodulation. IEEE Trans. Med. Imaging 39, 3391–3402 (2020). 32406828 10.1109/TMI.2020.2992498 PMC7717066

[26] M. Koltzenburg, C. L. Stucky, G. R. Lewin, Receptive properties of mouse sensory neurons innervating hairy skin. J. Neurophysiol. 78, 1841–1850 (1997). 9325353 10.1152/jn.1997.78.4.1841

[27] D. Dalecki, Mechanical bioeffects of ultrasound. Annu. Rev. Biomed. Eng. 6, 229–248 (2004). 15255769 10.1146/annurev.bioeng.6.040803.140126

[28] S. G. Lechner, G. R. Lewin, Hairy sensation. Physiology (Bethesda) 28, 142–150 (2013). 23636260 10.1152/physiol.00059.2012

[29] S. S. Ranade , Piezo2 is the major transducer of mechanical forces for touch sensation in mice. Nature 516, 121–125 (2014). 25471886 10.1038/nature13980 PMC4380172

[30] S. E. Murthy , The mechanosensitive ion channel Piezo2 mediates sensitivity to mechanical pain in mice. Sci. Transl. Med. 10, eaat9897 (2018). 30305457 10.1126/scitranslmed.aat9897 PMC6709986

[31] T. Hinoi , Mouse model of colonic adenoma-carcinoma progression based on somatic Apc inactivation. Cancer Res. 67, 9721–9730 (2007). 17942902 10.1158/0008-5472.CAN-07-2735

[32] B. P. Lehnert , Mechanoreceptor synapses in the brainstem shape the central representation of touch. Cell 184, 5608–5621.e18 (2021). 34637701 10.1016/j.cell.2021.09.023 PMC8556359

[33] C. Wetzel , A stomatin-domain protein essential for touch sensation in the mouse. Nature 445, 206–209 (2007). 17167420 10.1038/nature05394

[34] R. L. King, J. R. Brown, W. T. Newsome, K. B. Pauly, Effective parameters for ultrasound-induced in vivo neurostimulation. Ultrasound Med. Biol. 39, 312–331 (2013). 23219040 10.1016/j.ultrasmedbio.2012.09.009

[35] P. P. Ye, J. R. Brown, K. B. Pauly, Frequency dependence of ultrasound neurostimulation in the mouse brain. Ultrasound Med. Biol. 42, 1512–1530 (2016). 27090861 10.1016/j.ultrasmedbio.2016.02.012 PMC4899295

[36] E. Sassaroli, N. Vykhodtseva, Acoustic neuromodulation from a basic science prospective. J. Ther. Ultrasound 4, 17 (2016). 27213044 10.1186/s40349-016-0061-z PMC4875658

[37] W. J. Tyler, S. W. Lani, G. M. Hwang, Ultrasonic modulation of neural circuit activity. Curr. Opin. Neurobiol. 50, 222–231 (2018). 29674264 10.1016/j.conb.2018.04.011

[38] M. I. Brier, J. S. Dordick, Remote activation of cellular signaling. Science 368, 936–937 (2020). 32467375 10.1126/science.abb9122

[39] M. D. Menz, O. Oralkan, P. T. Khuri-Yakub, S. A. Baccus, Precise neural stimulation in the retina using focused ultrasound. J. Neurosci. 33, 4550–4560 (2013). 23467371 10.1523/JNEUROSCI.3521-12.2013 PMC6704938

[40] J. Kubanek, P. Shukla, A. Das, S. A. Baccus, M. B. Goodman, Ultrasound elicits behavioral responses through mechanical effects on neurons and ion channels in a simple nervous system. J. Neurosci. 38, 3081–3091 (2018). 29463641 10.1523/JNEUROSCI.1458-17.2018 PMC5864152

[41] Q. Gao, P. R. Cooper, A. D. Walmsley, B. A. Scheven, Role of piezo channels in ultrasound-stimulated dental stem cells. J. Endod. 43, 1130–1136 (2017). 28527849 10.1016/j.joen.2017.02.022

[42] M. L. Prieto, K. Firouzi, B. T. Khuri-Yakub, M. Maduke, Activation of Piezo1 but not Na_V_1.2 channels by ultrasound at 43 MHz. Ultrasound Med. Biol. 44, 1217–1232 (2018). 29525457 10.1016/j.ultrasmedbio.2017.12.020 PMC5914535

[43] D. Liao, M.-Y. Hsiao, G. Xiang, P. Zhong, Optimal pulse length of insonification for Piezo1 activation and intracellular calcium response. Sci. Rep. 11, 709 (2021). 33436695 10.1038/s41598-020-78553-2 PMC7804118

[44] S. Yoo, D. R. Mittelstein, R. C. Hurt, J. Lacroix, M. G. Shapiro, Focused ultrasound excites cortical neurons via mechanosensitive calcium accumulation and ion channel amplification. Nat. Commun. 13, 493 (2022). 35078979 10.1038/s41467-022-28040-1 PMC8789820

[45] Z. Qiu , The mechanosensitive ion channel Piezo1 significantly mediates in vitro ultrasonic stimulation of neurons. iScience 21, 448–457 (2019). 31707258 10.1016/j.isci.2019.10.037 PMC6849147

[46] Y. Pan , Mechanogenetics for the remote and noninvasive control of cancer immunotherapy. Proc. Natl. Acad. Sci. U.S.A. 115, 992–997 (2018). 29343642 10.1073/pnas.1714900115 PMC5798350

[47] S. E. Murthy , OSCA/TMEM63 are an evolutionarily conserved family of mechanically activated ion channels. eLife 7, e41844 (2018). 30382938 10.7554/eLife.41844 PMC6235560

[48] S. S. Ranade, R. Syeda, A. Patapoutian, Mechanically activated ion channels. Neuron 87, 1162–1179 (2015). 26402601 10.1016/j.neuron.2015.08.032 PMC4582600

[49] B. Sorum, R. A. Rietmeijer, K. Gopakumar, H. Adesnik, S. G. Brohawn, Ultrasound activates mechanosensitive TRAAK K^+^ channels through the lipid membrane. Proc. Natl. Acad. Sci. U.S.A. 118, e2006980118 (2021). 33542098 10.1073/pnas.2006980118 PMC8017979

[50] L. R. Gavrilov, G. V. Gersuni, O. B. Ilyinski, E. M. Tsirulnikov, E. E. Shchekanov, A study of reception with the use of focused ultrasound. I. Effects on the skin and deep receptor structures in man. Brain Res. 135, 265–277 (1977). 922476 10.1016/0006-8993(77)91030-7

[51] U. Andersson, K. J. Tracey, Neural reflexes in inflammation and immunity. J. Exp. Med. 209, 1057–1068 (2012). 22665702 10.1084/jem.20120571 PMC3371736

[52] J. P. Beekwilder, T. Beems, Overview of the clinical applications of vagus nerve stimulation. J. Clin. Neurophysiol. 27, 130–138 (2010). 20505378 10.1097/WNP.0b013e3181d64d8a

[53] H. Kahlow, M. Olivecrona, Complications of vagal nerve stimulation for drug-resistant epilepsy: A single center longitudinal study of 143 patients. Seizure 22, 827–833 (2013). 23867218 10.1016/j.seizure.2013.06.011

[54] M. G. Kim , Image-guided focused ultrasound modulates electrically evoked motor neuronal activity in the mouse peripheral nervous system in vivo. J. Neural Eng. 17, 026026 (2020). 31940596 10.1088/1741-2552/ab6be6 PMC7297566

[55] Animal Research Handbook, Columbia University. https://research.columbia.edu/sites/default/files/content/Animal%20Research%20Handbook%202020%20FINAL1.pdf. Accessed 12 May 2022.

[56] S. H. Woo , Piezo2 is required for Merkel-cell mechanotransduction. Nature 509, 622–626 (2014). 24717433 10.1038/nature13251 PMC4039622

[57] B. Coutaud, N. Pilon, Characterization of a novel transgenic mouse line expressing Cre recombinase under the control of the Cdx2 neural specific enhancer. Genesis 51, 777–784 (2013). 23913642 10.1002/dvg.22421

[58] S. A. Wellnitz, D. R. Lesniak, G. J. Gerling, E. A. Lumpkin, The regularity of sustained firing reveals two populations of slowly adapting touch receptors in mouse hairy skin. J. Neurophysiol. 103, 3378–3388 (2010). 20393068 10.1152/jn.00810.2009 PMC2888253

[59] B. U. Hoffman, E. A.E Lumpkin, Source Data For: “Focused ultrasound excites action potentials in mammalian peripheral neurons in part through the mechanically gated ion channel Piezo2.” https://doi.org/10.5281/zenodo.6513059. Deposited 2 May 2022. 10.1073/pnas.2115821119 PMC9173751 35580186

