# Supplementary material for: Focused ultrasound excites action potentials in mammalian peripheral neurons in part through the mechanically gated ion channel PIEZO2
Source: Proc Natl Acad Sci U S A. 2022 May 17;119(21):e2115821119. doi: 10.1073/pnas.2115821119 (PMC9173751; doi:10.1073/pnas.2115821119)
Supplement: Supplementary File [file pnas.2115821119.sapp.pdf]

PNAS
www.pnas.org

Supplementary Information for

Focused ultrasound excites action potentials in mammalian peripheral neurons in part through the mechanically gated ion channel PIEZO2

Benjamin U. Hoffman*, Yoshichika Baba*, Stephen A. Lee, Chi-Kun Tong, Elisa E. Konofagou‡, Ellen A. Lumpkin‡

*Equal contribution
‡Correspondence may be addressed to ellen.a.lumpkin@gmail.com or ek2191@columbia.edu

This PDF file includes:

Supplementary Methods
Figures S1 to S2
Tables S1 to S4

Supplementary Methods

Histological analysis of sonicated tissue. Tissue was sonicated as described previously (see *Methods*). The FUS parameter set employed was: stimulus duration (1 ms), intensity (743 W/cm^2, US frequency (3.57 MHz), and inter-stimulus interval (200 ms).

Sonicated samples were fixed with 10% formalin (Fisher Scientific, SF93-4 buffered Formalde-Fresh®) overnight, then transferred and kept in 70% ethanol for 24-48 hours. A Sakura VIP 5 tissue processor was used for serial dehydration as follows: Ethanol (70%, 1hr; 95%, 50min; 95%, 1hr; 100%, 1hr X 3); xyline, 1hr x 2; and paraffin, 1hr x 3 (Leica Microsystems™ Surgipath™ Paraplast™ Tissue Embedding Medium, Fisher Scientific: 23-021-751). Samples were paraffin embedded in a histology cassettes and cooled with Sakura Tissue-Tek® TEC™. Samples were sectioned at 5 µm with a Leica Microtome RM2255 and stained with H&E (Haemotoxylin Gill and Eosin) using a SAKURA Tissue-Tek DRS auto-stainer. Sections were mounted onto coverslips with mounting medium (Fisher premount Catalog# SP15-500).

Specimens were imaged with either a Leica DM6 B microscope (LAS X software) equipped with 20x/0.8 NA and 40x/0.95 NA Leica PL APO objectives, or a Zeiss Observer.Z1 microscope (Zen 2012 software) equipped with a 10x/0.50 NA (Zeiss Fluar), 20x/0.40 NA (Zeiss LD Plan-Neofluar Corr Ph2), and 40x/0.60 NA (LD Plan-Neofluar Corr Ph2) objectives. Images acquired at low magnification were stitched to generate representative images.

To generate epidermal and dermal+hypodermal thickness measurements, images were analyzed in ImageJ. Briefly, analysis windows were drawn around the estimated focal region of sonication ("FUS focus"), and two adjacent regions approximately 500–750 µm from the center of focus ("Left" and "Right"). Within each analysis window, the thickness of the skin compartment (epidermis or dermis+hypodermis) was measured six times. These measurements were averaged to estimate the mean thickness of each compartment. Two cryosections through the estimated focal point were analyzed for each sonicated preparation.

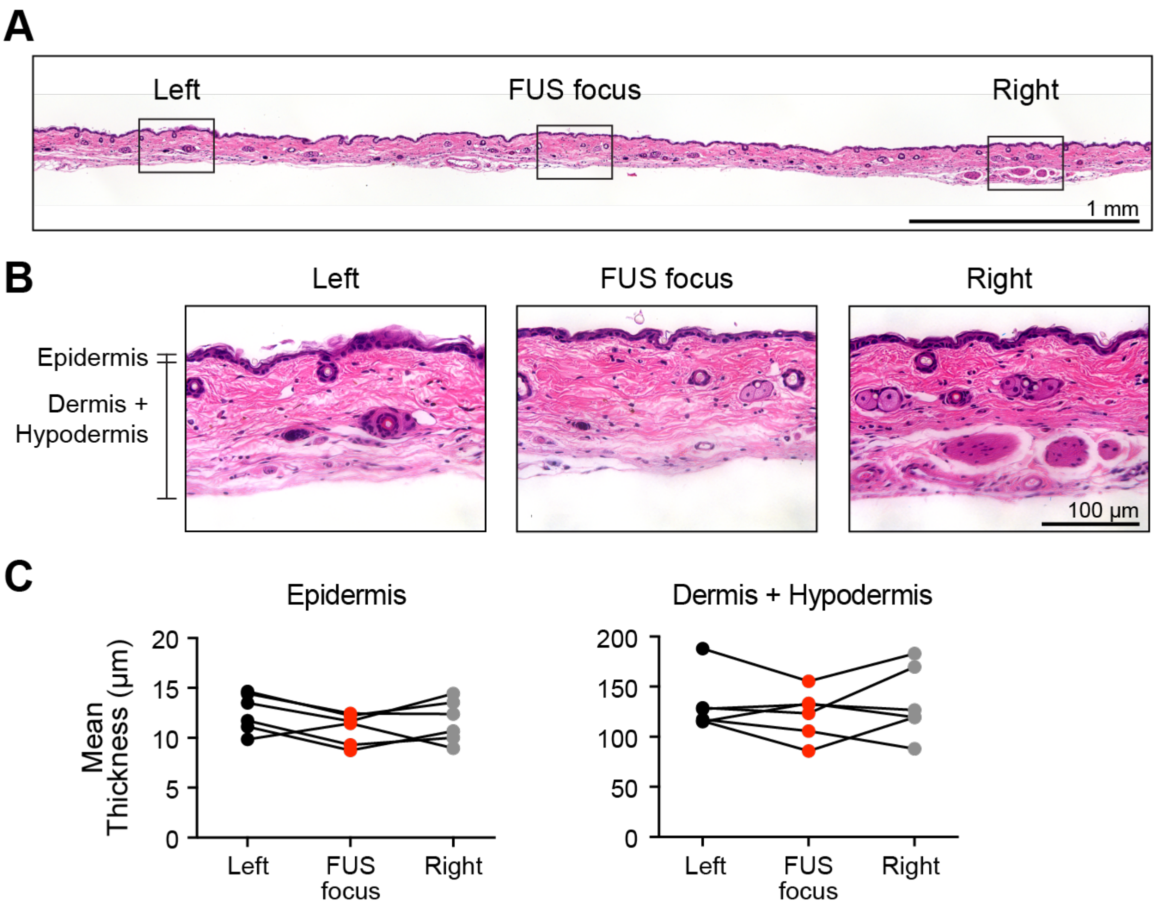

Fig. S1. High energy sonication does not cause histological damage, related to Fig. 2. **A.** Representative H&E cryosection of sonicated skin preparations. Target skin was sonicated 50 times at 0.2 Hz with 1301 nJ of ultrasound. Boxes represent analyzed regions. **B.** Magnification of regions identified in (A). Brackets indicate regions identified as epidermis and dermis+hypodermis. **C.** Analysis of epidermal and dermal+hypodermal thickness after sonication in targeted regions, compared to adjacent regions. Left, mean thickness of epidermis in analyzed regions. Right, mean thickness of dermis+hypodermis in analyzed regions. Lines, matched data from a single sonicated preparation. No significant differences were found (matched one-way ANOVA, *n*=6 preparations, 3 mice).

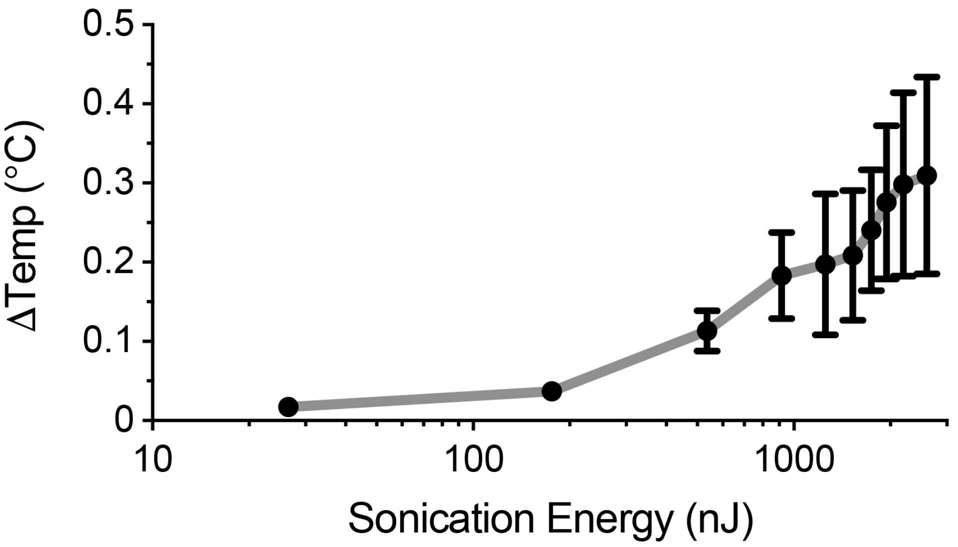

Fig. S2. FUS stimulation induces minimal local changes in temperature. Temperature measurements taken <1 mm from FUS focus area. A sequence of ten ultrasound stimuli were delivered (stimulus duration 2.0 ms; intensity, 40–743 W/cm^2, 25-60 W/cm^2 steps; US frequency, 3.57 MHz; inter-stimulus interval 5.0 s), displayed as sonication energy. The sequence was repeated teb times. Error bars denote SD; *n*=10 sequences. See *Materials and Methods* for detailed description for FUS parameters.

Table S1. Fiber characteristics, related to Figure 2.

	**Conduction velocity (m/s)**			**Mechanical Threshold (mN)**		
	Median	Quartile deviation	*n*	Median	Quartile deviation	*n*
**Aβ-RA**	13.1	1.6	25	0.7	0.7	25
**Aβ-SA**	12.6	2.7	30	0.2	0.2	30
**DH**	7.3	2.2	35	0.2	0.2	35
**AM**	6.7	1.7	47	1.6	1.7	47
**C**	0.4	0.1	35	1.6	2.4	22

Table S2. Stimulus-response fits, related to Figure 2.

	Top (probability)	Slope	EC_{50} (nJ)	Bottom (probability)	R^2	$Energy_{50\%Prob}$ (nJ)
**Aβ-RA**	0.99	1.14	232	0	0.66	233
**Aβ-SA**	0.86	1.68	146	0	0.73	177
**DH**	0.97	1.12	47	0	0.66	50
**AM**	0.77	1.13	160	0	0.41	280
**C**	0.78	1.09	133	0	0.60	225

Table S3. Stimulus-response fits, related to Figure 6.

	**Top**	**Slope**	**EC_{50}**	**Bottom**	**R^2**	**$E_{50\%Respond}$**
**Mechanical: Control**	0.87	0.98	0.6 mN	0	0.97	0.8 mN
**Mechanical: *Cdx2^{Cre};Piezo2$^{fl/fl}$***	0.75	1.23	6.7 mN	0	0.99	11.5 mN
**FUS: Control**	0.95	2.17	151.7 nJ	0	0.99	158.5 nJ
**FUS: *Cdx2^{Cre};Piezo2$^{fl/fl}$***	0.77	2.12	333.8 nJ	0	0.97	447.5 nJ

Table S4. US transducer calibration table.

Input Voltage (mV)	Peak Positive Pressure (MPa)	Peak Negative Pressure (MPa)	Pulsed Average Intensity (W/cm^2)
10	0.2	-0.2	0.1
20	0.3	-0.3	0.6
30	0.4	-0.4	3.5
40	0.6	-0.4	6.1
50	0.7	-0.5	9.4
60	0.9	-0.6	13.7
70	1.1	-0.7	19.1
80	1.2	-0.7	24.9
90	1.5	-0.8	32.4
100	1.7	-0.9	39.8
110	2.0	-1.0	48.5
120	2.3	-1.0	57.9
130	2.7	-1.1	69.4
140	3.2	-1.1	81.9
150	3.6	-1.2	95.1
160	4.0	-1.2	108.9
170	4.3	-1.3	128.2
180	4.6	-1.3	141.2
190	4.6	-1.4	144.2
200	4.8	-1.4	163.2
210	5.1	-1.5	167.7
220	5.2	-1.6	181.2
230	5.6	-1.6	203.1
240	5.6	-1.7	216.6
250	6.0	-1.8	233.4
260	6.1	-1.8	236.1
270	6.2	-1.9	249.0
280	6.4	-1.9	260.2
290	6.5	-1.9	266.2
300	6.8	-1.9	267.8
310	6.9	-2.0	300.1
320	6.8	-2.0	317.2
330	6.9	-2.1	322.2
340	7.0	-2.1	345.4
350	7.0	-2.1	345.5
360	7.4	-2.2	346.5
370	7.5	-2.2	350.4
380	7.2	-2.2	346.9
390	6.9	-2.2	367.4
400	7.0	-2.3	374.1
410	7.1	-2.3	376.8
420	7.2	-2.4	413.7
430	7.2	-2.3	396.4
440	7.5	-2.4	393.4
450	7.6	-2.4	405.5
460	7.7	-2.5	416.2
470	7.6	-2.5	424.5
480	7.8	-2.4	428.7
490	7.6	-2.5	451.1
500	8.5	-2.5	486.0
510	8.1	-2.5	431.0

520	7.4	-2.5	573.7
530	7.3	-2.6	453.3
540	7.6	-2.5	483.4
550	7.5	-2.5	485.9
560	7.7	-2.6	501.1
570	8.4	-2.6	510.4
580	8.8	-2.6	575.6
590	7.4	-2.6	494.1
600	8.2	-2.7	544.9
610	8.5	-2.6	469.1
620	8.6	-2.7	576.1
630	7.9	-2.6	499.5
640	8.7	-2.7	496.7
650	9.0	-2.7	606.3
660	9.0	-2.7	605.0
670	8.1	-2.7	539.9
680	9.0	-2.8	587.3
690	9.2	-2.7	511.6
700	8.7	-2.8	609.5
710	8.9	-2.8	582.0
720	8.7	-2.8	528.6
730	9.0	-2.8	543.7
740	9.1	-2.9	644.3
750	8.9	-2.8	532.4
760	9.3	-2.8	666.9
770	9.2	-2.7	595.2
780	9.3	-2.8	606.4
790	9.3	-3.0	670.5
800	10.0	-3.0	762.6
810	10.0	-3.3	783.8
820	9.6	-3.0	746.3
830	10.1	-2.1	778.5
840	10.2	-3.0	719.2
850	9.4	-3.0	695.4
860	9.6	-3.0	699.1
870	9.3	-3.0	684.7
880	10.3	-3.0	795.4
890	10.2	-3.3	672.7
900	10.1	-3.0	773.6
